# Coherent Excitation of the CH Stretching Vibrations in C_2_H_4_ ^+^: The Role of the Derivative Coupling Studied by the Quantum Ehrenfest Method

**DOI:** 10.1002/jcc.70028

**Published:** 2025-01-11

**Authors:** Thierry Tran, Graham A. Worth, Michael A. Robb

**Affiliations:** ^1^ Nantes University, CNRS, CEISAM UMR 6230 Nantes France; ^2^ Department of Chemistry University College London London UK; ^3^ Department of Chemistry Molecular Sciences Research Hub, Imperial College London London UK

**Keywords:** attochemistry, nonadiabatic dynamics, quantum dynamics

## Abstract

We report nonadiabatic dynamics computations on C_2_H_4_^+^ initiated on a coherent superposition of the five lowest cationic states, employing the Quantum Ehrenfest method. In addition to the totally symmetric carbon–carbon double bond stretch and carbon‐hydrogen stretches, we see that the three non‐totally symmetric modes become stimulated; torsion and three different C—H stretching patterns. Thus, a coherent superposition of states, of the type involved in an attochemistry experiment, leads to the stimulation of specific non‐totally symmetric motions. The computations were also performed on the specific combination of the A and C states. In each case normal mode 9 (cis‐asymmetric H_2_CCH_2_ stretch), out of the set of non‐totally‐symmetric normal modes, dominates. Thus, we can steer the nuclear motion along specific non‐totally symmetric normal modes using a defined coherent superposition.

## Graphical abstract

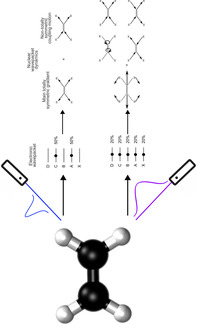

## 1 Introduction

The emergence of attosecond laser sources [1, 2, 3, 4] gives one the opportunity to probe molecular systems on the timescale of the electronic motion. One main property of attosecond laser experiments is that the high energy photon can lead to photoionization from the whole of the laser energy bandwidth, leading to potential ionization/excitation of multiple electronic states in a coherent manner. From a theoretical perspective, the electronic wavepacket created from a coherent superposition of states exhibits new properties. To simulate the molecular evolution, two strategies can be followed. One is purely focused on the electron dynamics and is obtained by propagating the electronic wavepackets in time on a frozen molecular frame [5]. The second is the simulation of the full molecular wavefunction to assess the coupled electron‐nuclear dynamics stemming from a coherent superposition. Different theoretical approaches can be found in the literature for the study of the interplay between electronic coherence and nuclear motion such as in the work of Vismarra et al. on donor–π–acceptor molecules [6], on attosecond delays in molecules by Suñer‐Rubio et al. [7] and in a study of Despré et al. on attosecond hole migration in benzene [8].

Alternatively, the superposition of electronic states can be treated in an incoherent manner by averaging the results obtained from dynamics initiated on each individual state. In this case, one can perform the dynamics using the Tully surface hopping method [9, 10, 11, 12, 13] starting with some appropriate sample of nuclear configurations. In the surface hopping method, the effect of the derivative coupling on the nuclear motion is treated in an ad hoc fashion by correcting the momentum vector along the derivative coupling direction at a hopping event. In this method, the contribution of the derivative coupling stemming from an electronic wavepacket is not included in the gradient and thus it doesn't impact the motion of the trajectories outside of hopping event or only as a single event correction when switching states for energy conservation purpose. Thus, the method is not suitable for attochemistry modeling [14].

In the case of a coherent superposition, the initial electronic state must be defined as an electronic wavepacket, requiring methods that can properly describe the coupled electron‐nuclear dynamics induced by a coherent superposition. Here one starts with the time‐dependent Schrödinger equation (TDSE) and computes the electron dynamics. One then computes the gradient of the resulting electronic wavepacket at each timestep for the nuclear dynamics [15] (see also exact factorization [16, 17]). This strategy then gets further extended by expressing the nuclear wavepacket as an expansion of interacting nuclear basis functions (e.g., Gaussian wavepackets [GWP]). A non‐exhaustive list of nonadiabatic dynamics methods that fall within this category are MCTDH [18], Multiconfigurational Ehrenfest [MCE] [19] and DD‐vMCG [20] (and its Ehrenfest equivalent Quantum Ehrenfest [QuEh]) [21].

The important distinction between the various approaches (i.e., TDSE for electronic and nuclear motion vs. surface hoping based on adiabatic surfaces) lies in the gradient that drives nuclear motion, in particular the derivative coupling [14]. We have discussed the effect of these cross‐mixing terms in depth elsewhere [22, 23, 24]. For the Ehrenfest‐based approach, one might use the average gradient of the states in a superposition (so called “mean field” Ehrenfest). However, this ignores the fact that the coherent superposition is a unique state, distinct from its components. The alternative methodology is to treat the electronic wavepacket as a proper state and obtain the full expressions for the gradient and Hessian.

For the current work, we are using the QuEh method with full second order expansion of the Ehrenfest state from the work of Vacher et al. [15]. Recently we have shown that this approach can be used to study the dynamics of a localized hole [25, 26, 27] which gives rise to coherences and also to study passage through a conical intersection where coherences are generated at the crossing [28].

For nonadiabatic dynamics based on GWP, the propagation of the nuclear basis functions can be separated into two parts: the expansion coefficient from which the weight (i.e., using the Gross Gaussian [29] population to account for non‐orthogonality of the GWP) can be extracted and the Gaussian parameters (position, momentum and phase of the center of the GWP). The former component is treated quantum mechanically by solving the TDSE with the GWP ansatz. The latter component can be treated purely classically (i.e., classical trajectory) by simply evolving the center of the GWP according to Newton equation‐of‐motion in methods such as Ab Initio Multiple Spawning [30, 31] and the first version of MCE [19]. The recent work of Mattos et al. with a post‐analysis approach to Surface Hopping can be analogous to propagating GWP along classical trajectories [32]. In the case of DD‐vMCG (and QuEh), the GWP follow a “quantum” trajectory by obtaining the equation‐of‐motion for the Gaussian parameters by applying the Dirac‐Frenkel variational principle [33] to the TDSE. We refer to the review on the vMCG method for more details [20].

The ethylene (ethene in UPAC naming nomenclature) cation is the simplest carbon π radical system and has been extensively studied from both an experimental and theoretical approaches [34, 35, 36, 37, 38, 39, 40, 41, 42, 43, 44, 45, 46, 47].

The five lowest cationic states are D_0_: X, $\pi^{+}$, B_3u_, D_1_: A, $\sigma {\left(C-H\right)}^{+}$, B_3g_, D_2_: B, $\sigma {\left(C-C\right)}^{+}$, A_g_, D_3_: C, $\sigma {\left(C-H\right)}^{+}$, B_2u_, and D_4_: D, $\pi^{*+*}$, B_1u_. In subsequent discussion we will use the spectroscopic notation X, A, B, and so forth, for simplicity. The ground cationic state X has a non‐planar geometry with two close local minima separated by a low torsional barrier [35, 36]. Upon excitation to higher cationic states (D_1_ and above), the molecules have been shown to subsequently fragment leading to a H‐ or H_2_‐loss with the specific fragment observed dependent on the photon energy used for ionization [34, 46]. Various decay channels are mediated by a conical intersection through either a pyramidalization or isomerization structure and have been highlighted by previous nonadiabatic dynamics simulations in the cationic manifold with a potential link between the fragmentation product and the decay channel [41, 43, 47]. Previous work focuses on the photochemistry associated with individual cationic states and with the emergence of attosecond lasers, more recent work has investigated the effect of ionizing to multiple cationic states with an incoherent superpositions [43, 46]. Here, we shall discuss the nuclear dynamics that occurs if one were to create a coherent superposition of all five low‐energy states of the ethylene cation C_2_H_4_^+^. In the experiments of Lucchini et al. [46], the same five states were excited with a broad energy laser pulse but there is an open question as to whether a coherent electronic wavepacket has been created under these experimental conditions.

Thanks to the high symmetry of the ethylene molecule (D_2h_), the dynamics can be rationalized in terms of symmetry rules. The gradient of an adiabatic state must always be totally symmetric. However, for a coherent superposition the situation is different. The gradient (or force) that drives the nonadiabatic dynamics of a coherent superposition has two types of component: intrastate and interstate. The later (off‐diagonal gradients that arise from the mixing) are the derivative couplings and have the form $<\psi_{I}\left|\partial /\partial Q_{i}^{\alpha_{i}}{\hat{H}}_{e}\right|\psi_{\mathrm{II}}>$ where the I and II are two adiabatic states and $\partial /\partial Q_{i}^{\alpha_{i}}$ is the gradient operator for each normal mode *i* with symmetry $\alpha_{i}$. As just stated, the gradient of the intrastate terms is only non‐zero along normal modes belonging to totally symmetric irreducible representations for the interstate terms, the mixing/superposition of two states I and II will be “allowed”, with an off‐diagonal gradient component along $Q_{i}^{\alpha_{i}}$, only if $\alpha^{I}⊗\alpha^{Q_{i}^{\alpha_{i}}}⊗\alpha^{\mathrm{II}}=A_{1g}$ (A_1g_ is the totally symmetric representation in the D_2h_ symmetry point group) and α is an irreducible representation label. Thus, for a coherent superposition we can expect to see initial motion along non‐totally symmetric coordinates.

We should emphasize at this point that there is only a single time‐dependent potential surface associated with each Gaussian wavepacket. Thus, the population transfers we will discuss subsequently in Figure 4 do not truly represent a passage through a conical intersection. We have discussed the relationship of QuEh with a conical intersection elsewhere [24]. The issue has also been discussed in some detail by Curchod and Agostini [48] in relation to time‐dependent potential energy surface. As we point out subsequently in this paper, exact factorization and QuEh differ mainly in the fact that in the latter we expand the nuclear wavefunction using GWP.

To clarify the concept of symmetry in the case of ethylene cation, Table 1 presents the irreducible representations resulting from the direct product for each pair of electronic states, along with the corresponding NM that share the same irreducible representation. An electronic wavepacket composed of a specific pair of electronic states will induce a motion along the derivative coupling direction on the nuclear wavepacket. For example, mixing states of symmetry B_3g_ with B_2u_ (for states A and C, respectively) results in derivative coupling motion with irreducible representation B_1u_ corresponding to NM9.

**TABLE 1:** Symmetry (direct) product (which we subsequently refer to using the direct product notation ⊗) of the pair of electronic states and the resulting NM associated with the same irreducible representation (i.e., interstate gradient).

	X (B_3u_)	A (B_3g_)	B (A_g_)	C (B_2u_)	D (B_1u_)
X (B_3u_)	A_g_	A_u_ NM 4 (torsion)	B_3u_	B_1g_	B_2g_
A (B_3g_)		A_g_	B_3g_ NM 11	B_1u_ NM 9	B_2u_ NM 12
B (A_g_)			A_g_	B_2u_ NM 12	B_1u_ NM 9
C (B_2u_)				A_g_	B_3g_ NM 11
D (B_1u_)					A_g_

**FIGURE 1:**
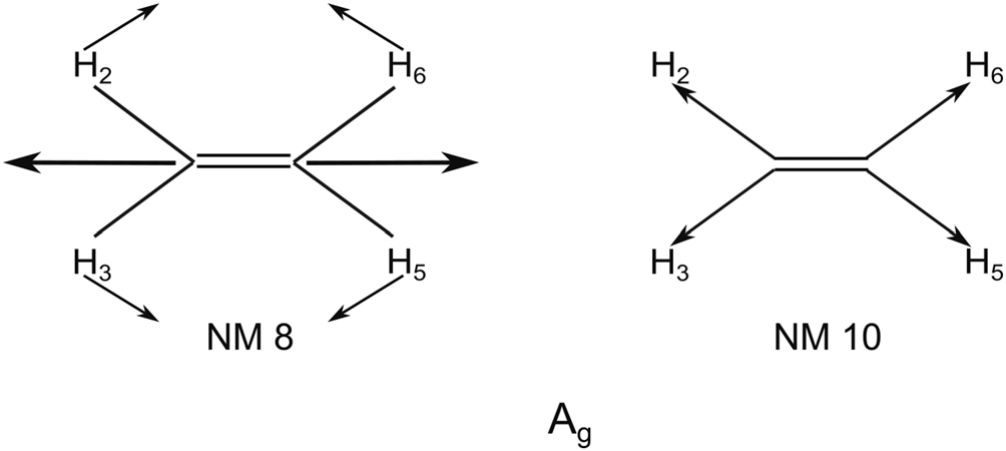
The two totally symmetric NM included in the reduce dimension for the nonadiabatic dynamics. NM8 is the carbon–carbon stretching and NM10 is the symmetric stretching of all four carbon‐hydrogen bonds.

**FIGURE 2:**
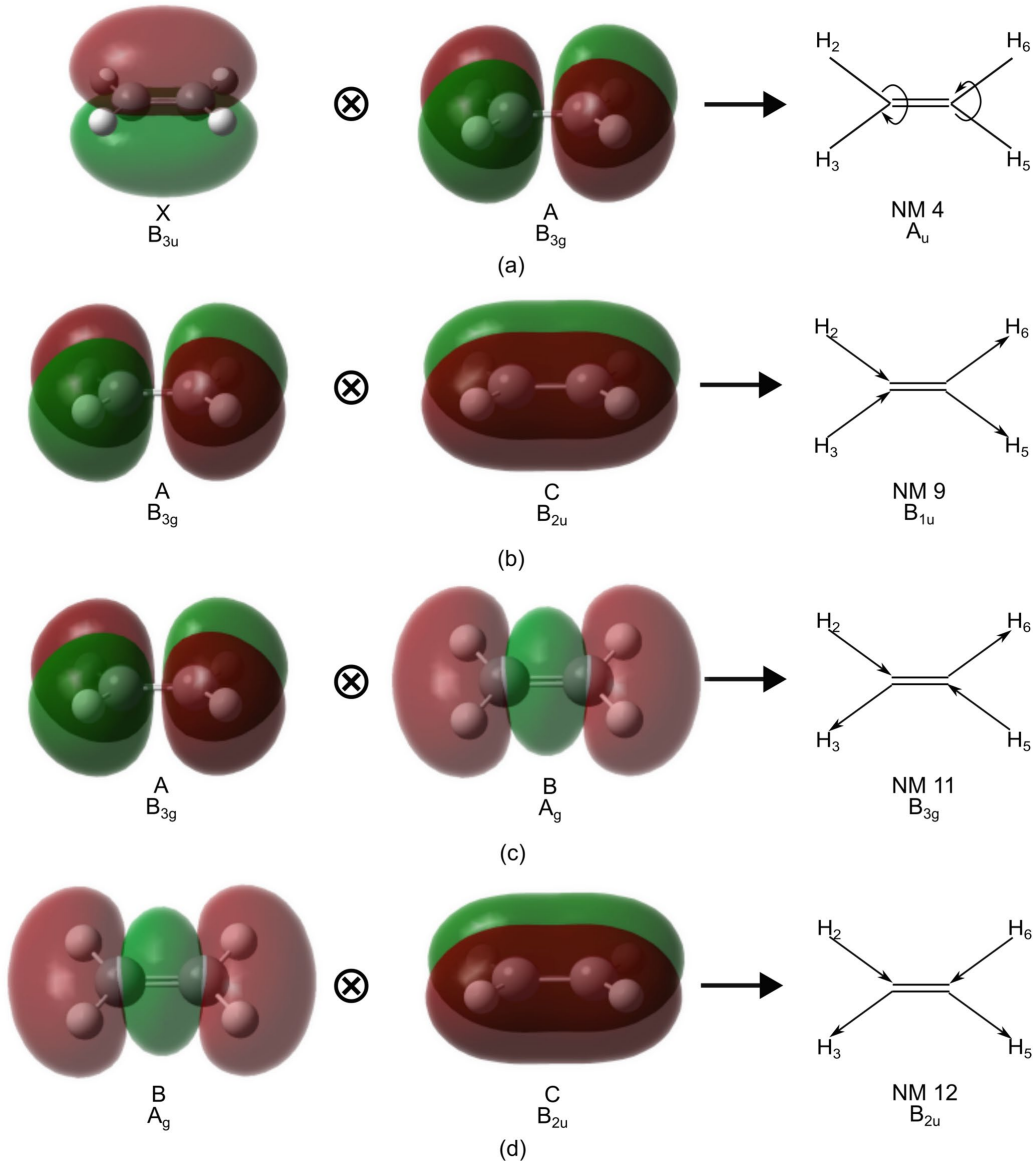
Non‐totally symmetric NM corresponding to the torsional motion and carbon‐hydrogen bond stretching, along with the singly occupied molecular orbital associated with the pair of electronic states satisfying the direct symmetry product to yield the corresponding irreducible representation.

Thus, the goal of this paper is to examine the initial nuclear dynamics of a coherent superposition of all five cationic states. The dynamics are performed in reduced dimension in the space spanned by the main totally symmetric motions (see Figure 1) and the relevant NM related to the coupling mode for the states included in the dynamics (see Figure 2). The results are analyzed by symmetry selection rules with respect to the derivative coupling. We shall see that the derivative coupling stimulates non‐totally symmetric motion.

Since we cannot know the extent of the coherence in these superpositions or the phase upon photoionization with an attosecond laser in experiment, our simulations give examples of an idealized fully coherent superposition where all five states have the same sign and phase initially. Thus, in our computations we arbitrarily choose all the signs to be positive in the coherent superposition. From the experimental data we can only get information about the relative weight between states using the ionization cross‐sections. The phase between states could be determined theoretically by explicitly including the ionization step in the dynamics with a presence of a pulse [49, 50]. The alternative in a sudden ionization approach for dynamics is to calculate separately the initial electronic wavepacket created upon photoionization from a specific attosecond pulse with the method of Ruberti et al. [51].

## 2 Theoretical Background

The equation‐of‐motion for the QuEh method [52] can be obtained by using the exact factorization ansatz for the TDSE [16, 17].

(1) $$\Psi \left(R,r,t\right)=\chi \left(R,t\right)\psi \left(r,t;R\right)$$

where the molecular wavefunction Ψ is written as a product of nuclear χ and electronic wavefunction ψ with the parameters **R** denoting the nuclear position, **r** the electron position and *t* the time. In QuEh, the nuclear wavefunction is expanded as a sum of GWPs *g*_*j*_ with expansion coefficient *A*_*j*_.

(2) $$\Psi \left(R,r,t\right)=\sum_{j}A_{j}\left(t\right)g_{j}\left(R,t\right)\psi \left(r,t;R_{j}\right)$$

The equations‐of‐motion of the expansion coefficients *A*_*j*_ and GWP parameters (position, momentum and phase) are obtained from solving the TDSE using the Dirac‐Frenkel variational principle. For more details on the equation‐of‐motion for the GWP, information can be found on the work of Richings et al. [20] and in the current work, we will briefly focus on the propagation of the electronic wavepacket.

Starting from exact factorization enables the definition of a global time‐dependent “Ehrenfest” potential surface which means that the expansion coefficients of the nuclear wavepacket do not depend directly on a time‐independent electronic state as in the Born‐Huang expansion. The approximation then made is that the Ehrenfest surface is only known around the center of each GWP (denoted by the R_*j*_ as parameter of the electronic wavefunction) using a local harmonic approximation. QuEh equations‐of‐motion can also be obtained starting from the Born‐Huang ansatz [53] in which each GWP is associated with a local electronic wavepacket. This, however, introduces couplings between the electronic wavepackets which are difficult to compute.

The electronic wavepacket around R_j_ can be expanded locally (denoted by the index *j* in $\psi_{j}$) into a sum of individual electronic states, here chosen as eigenvectors of the electronic Hamiltonian at that point.

(3) $$\psi_{j}\left(r,t;R_{j}\right)=\sum_{s}c_{s,j}\left(t\right)\psi_{s}\left(r,R_{j}\right)$$

The electronic wavepacket is propagated locally in time using the Ehrenfest method [52] with the equation‐of‐motion of the electronic state complex coefficients given by the following expression.

(4) $$i{\dot{c}}_{s,j}\left(t\right)=\sum_{s^{\prime }}H_{ss^{\prime }}^{\mathrm{el}}\left(R\right)c_{s^{\prime },j}\left(t\right)$$

By construction, the electronic wavepacket is built as a coherent superposition of electronic adiabatic states and directly impacts the motion of the associated GWP. A feature of our implementation of the QuEh is that the full derivative coupling is included in the expression for the analytical gradient [15] experienced by the nuclear basis function so it should not be regarded as a mean field approach. The gradient (or force) that drives the nonadiabatic dynamics of a coherent superposition has two types of components: intrastate and interstate. Thus, the dynamics that arises from the mixing of two states is very similar to the forces that act at a conical intersection where one force component is directed along the derivative coupling. Indeed, one may make a prediction about the course of the nuclear dynamics as a result of state mixing by computing the derivative coupling as we shall presently show.

The expectation value of any operator $\hat{o}$ can be approximated as a sum of quantities evaluated at the center of each GWP, *o*_*j*_, weighted by its Gross Gaussian Population (GGP) to properly account for the non‐orthogonality of the Gaussian function.

(5) $$<o\left(t\right)>=<\Psi \left(t\right)\left|\hat{o}\right|\Psi \left(t\right)>\approx \sum_{j}{\mathrm{GGP}}_{j}\left(t\right)o_{j}\left(t\right)$$

(6) $${\mathrm{GGP}}_{j}\left(t\right)=ℜ\left\{\sum_{i}S_{\mathrm{ij}}\left(t\right)A_{i}^{*}\left(t\right)A_{j}\left(t\right)\right\}$$

## 3 Computational Details

The dynamics is propagated for 20 fs with the QuEh method implemented in QUANTICS [54] and interfaced with a development version of the Gaussian quantum chemistry software [55] where the electronic motion is represented using a complete active space configuration interaction (CAS‐CI) formulation for the Ehrenfest method with a 6‐31 g* basis set. The active space consists of 11 electrons in seven orbitals (the two carbon π orbitals, the four carbon‐hydrogen σ bond orbitals and the carbon–carbon σ bond orbital). The integrator for the nuclear dynamics is Runge–Kutta fifth order. The dynamics is performed in reduced dimension in NM coordinates defined as mass and frequency scaled NM vector from a ground state S_0_ Franck‐Condon point frequency calculation at 6‐31 G*/B3LYP level of theory. The reduced dimension comprises six NMs out of the full 12 and includes the torsional motion (NM4), the symmetric carbon–carbon bond stretching (NM8), and all four NMs related to the carbon‐hydrogen bond vibrations (NM9, 10, 11 and 12).

The initial nuclear wavefunction is represented as a linear combination of 13 GWPs in a “full shell” sampling in momentum space using a local harmonic approximation along the defined NM coordinate. The “full shell” has 2n + 1 GWPs with n corresponding to the number of nuclear degrees of freedom: All GWP initial positions are at the Franck‐Condon geometry with the first GWP possessing no initial momentum, GWP 2 to 2n + 1 have an initial momentum along a single NM with either a positive and negative value so as to make an overlap of 0.7071 between neighboring GWPs (i.e., a pair of GWPs are set up for each NM). To ensure a stable nonadiabatic dynamics with QuEh, the width of the GWPs and initial nuclear wavepacket along every degree of freedom (i.e., NM) is set to 0.24 and 0.35, respectively.

In the mass‐frequency scaled normal mode coordinates used for the QuEh simulations, the ground‐state vibrational wavefunction in the harmonic approximation of the ground‐state potential is a Gaussian function with a width of 0.7071 ($\frac{1}{\sqrt{2}}$) (in arbitrary unit).

In on‐the‐fly nonadiabatic dynamics with QuEh, one can suffer from numerical instability due to the numerical integration of the equations‐of‐motion. The integration of the nuclear equation‐of‐motion involves the evaluation of the inverse of the overlap matrix between GWPs. At the beginning of the dynamics, the GWPs are all located closely in phase space which could lead to a singular overlap matrix which results in a loss of the conservation of the total energy of the system. The widths of the GWP and the initial nuclear wavefunction were chosen to reduce the possibility of obtaining a non‐invertable overlap matrix, thus giving a more reliable total energy conservation. The consequence of a choice of width different from the standard value of 0.7071 leads to an excited vibrational wavepacket.

The dynamics can be initiated on different superpositions of electronic states by choosing specific coefficients for each cationic adiabatic state where the initial configuration state function vectors are computed at the Franck‐Condon point. Since our objective is to understand the role of the derivative coupling in a coherent superposition we have chosen equal initial weights of each of the five lowest cationic states: X, A, and so forth. In addition, we have run a simulation for the coherent superposition of states A and C.

## 4 Results and Discussion

**FIGURE 3:**
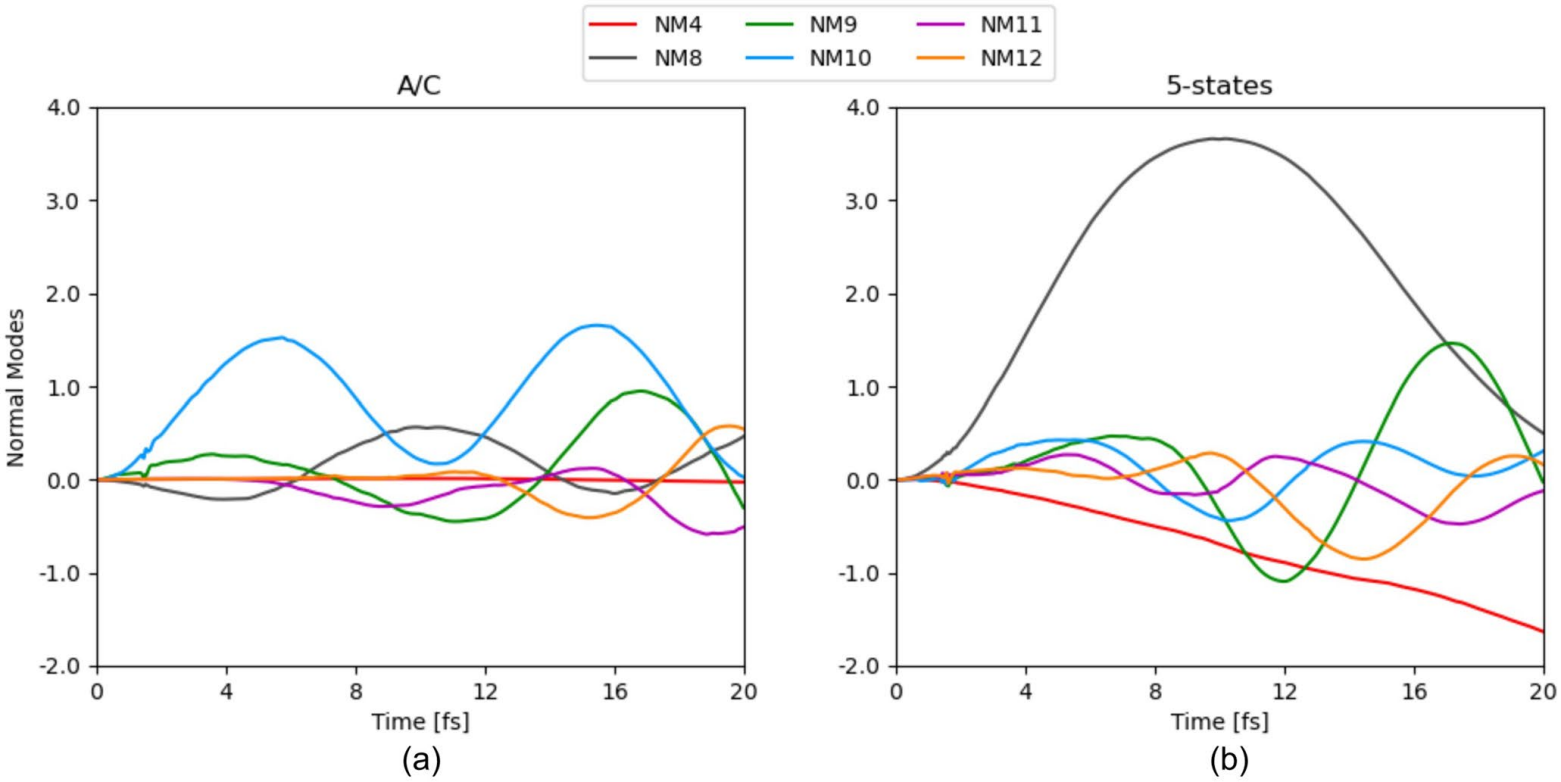
(a) Expectation value of the normal modes for the nuclear dynamics of (a) a 2 state superposition (A and C) and (b) for the five state superposition.

In Figure 3a we show the expectation value of the NM motion for the nuclear wavepacket initiated on a coherent superposition of two (A/C) of the five cationic states X, A, and so forth. and Figure 3b on a superposition of all the five cation states weighted equally. We only display the results for a few NM as they are the most relevant for the previously found excited state decay pathways as well as from a symmetry perspective based on the nature of the states involved. We can see that the totally symmetric motion such as the carbon–carbon bond stretching (NM8) and symmetric stretching of all four carbon‐hydrogen bonds (NM10) have large amplitudes. Moreover, the torsional (NM4), cis‐ (NM9) and trans‐asymmetric (NM12) motion are also stimulated in the dynamics initiated on a coherent superposition of all five states (see Figure 3b). In the case of a simpler system with an electronic wavepacket composed of only two states, it is expected that the nuclear wavepacket will be driven by motion with the irreducible representation satisfying the symmetry product rule. In other words, the gradient driving the dynamics is the sum of the adiabatic state gradients and the derivative coupling. By looking at Figure 3a where the dynamics is initiated on a coherent superposition of state A and C with B_3g_ and B_2u_, the symmetry allowed motion are the totally symmetric component and the derivative coupling of B_1u_ irreducible representation which is NM9 in this case. Moreover, the hole created in the A and C states is located on carbon‐hydrogen σ molecular orbitals and will weaken the bond leading to a major contribution of NM10 into the nuclear wavepacket motion.

By comparison with the five state superposition, we can observe a major contribution of NM9 to the motion of the center of the nuclear wavepacket due to the presence of the A and C states in the initial electronic wavepacket. Furthermore, the idea can be further expanded by taking all pair of states. From Figure 2, the nuclear motion is expected to be steered along NM11 and NM12 as well from taking the contribution from the other pair of states involved in the superposition and the expectation values of the NM displacement of the nuclear wavepacket do move along the derivative coupling direction associated to the other pair of electronic states included into the initial electronic wavepacket (see Figure 3b).

Another observation that can be made is the large contribution of the carbon–carbon double bond stretching (NM8) to the nuclear dynamics initiated on a coherent superposition of all five cationic states. The weakening of the double bond can be explained by the presence of the B state in the superposition which at the Franck‐Condon point corresponds to creating a hole in the carbon–carbon σ bond. After 20 fs, on top of the NM8 contribution, the dynamics is dominated by NM9 stemming from the A/C derivative coupling contribution as well as the torsional motion due to the X/A coupling motion. From previous theoretical work [43], the torsional motion is one of the main deactivation channels for excited state decay in ethylene cation.

**FIGURE 4:**
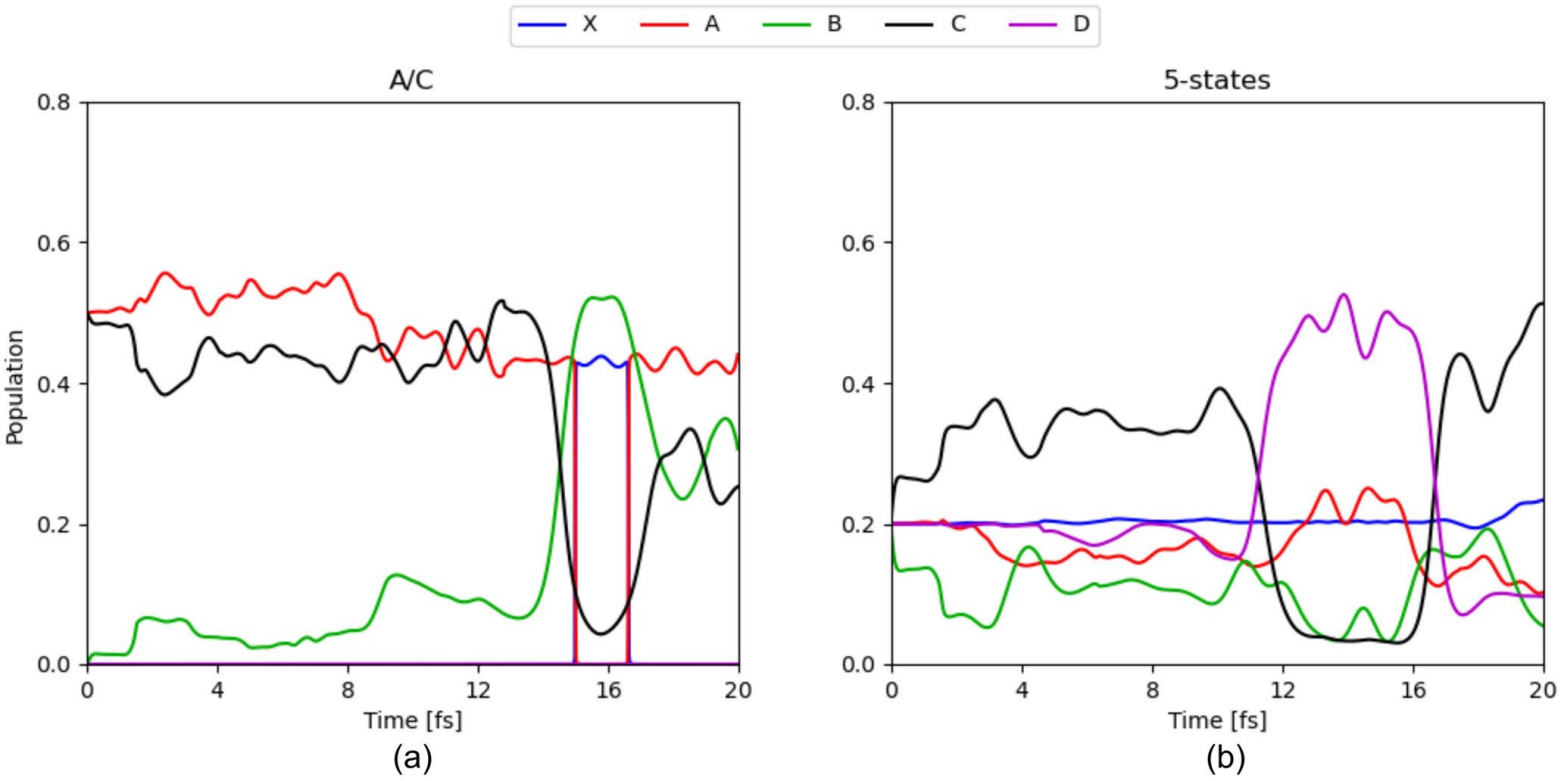
Adiabatic state populations (a) for A/C superposition (red and black, respectively) and (b) for the five‐state superposition.

By focusing on the electron dynamics indicated by the adiabatic state population in Figure 4, oscillating electron dynamics are observed between state A and C when initiating the nonadiabatic dynamics with a coherent superposition of these two states. It is known from previous theoretical work that a coherent superposition of two electronic states induces oscillating electron dynamics with the time period inversely proportional to the energy gap between the two states [56]. Up to approximately 12 fs, a gradual step‐wise increase of the population on the state B is noted, which can be rationalized by its energetic location between states A and C. After 12 fs, a rapid population transfer to the B occurs, which is characteristic of a nuclear wavepacket going through a conical intersection.

Initiating the dynamics on a coherent superposition of all five lowest cationic states (see Figure 4b) leads to a more complicated picture. For the first half of the dynamics, the electron dynamics is mainly driven by the population transfer between states B and C. The second half is dominated by large population transfer which point toward the presence of conical intersection along the pathway of the nuclear wavepacket dynamics with an initial transfer to the highest cationic state D followed by a recovery of the population on the C states. For the 20 fs of the simulation, the dynamics is controlled by the high energy cationic states and we only observe a gradual increase to the ground cationic state X around 18 fs.

As we have discussed above, the changes in adiabatic state populations (e.g., Figure 4a) at 16–17 fs are cases where the diabatic state remains the same but the ordering in the adiabatic representation changes.

In QuEh, there is only one time‐dependent potential energy surface for each GWP so a change in the electronic coefficient is not associated with a full discontinuous change of the potential energy surface which would be typical in surface hopping method. Rather they merely reflect the change in the composition of the coherent superposition with time and emphasize that the coherent superposition is a “state” in itself with its own gradient and the nuclear dynamics is driven by the first derivative of the time‐dependent Ehrenfest state. The stability of the nonadiabatic dynamics with QuEh makes it challenging to extend the simulation for longer time on top of the computational cost. Thus, in the current work, we can only predict the expected outcome of the nuclear wavepacket dynamics induced by electronic states that can potentially be photoionized to in a coherent manner from recent experimental work with large energy bandwidth laser [46].

## 5 Conclusions

For attochemistry simulations, where a coherent electronic superposition is created, it is important to properly account for the effect of the derivative coupling on the nuclear wavepacket evolution. Here we show that a specific mixing of two states (A/C) generates large amplitude motion along the symmetric carbon‐hydrogen stretching NM10 and the associated derivative coupling NM9 whereas a mixing of all five states leads to a contribution of multiple derivative couplings terms steering the nuclear dynamics as well as a weakening of the central carbon–carbon double bond showcased by the large amplitude motion along NM8. Thus, by carefully creating a specific initial electronic wavepacket, it is possible to achieve a form of coherent control.

In the recent experimental and theory paper [46], superpositions were created from three different harmonics of a high‐harmonic generation laser source (H9, H11 and H13) and a photoionization with the ninth harmonic result in an electronic wavepacket dominated by the X and A states. For the other two harmonics mentioned in the experimental work, the 11th and 13th lead to an ionization mainly into the A, B and C states. The main experimental results were the fragmentation pattern C_2_H_3_^+^ versus C_2_H_2_^+^. However, under their experimental conditions the superposition would not have been coherent. In our results corresponding to a coherent excitation with five states we see an initial double CH stretch (cis NM 12 trans NM 11 and sym‐CH_2_ (NM 9) $H_{2}\leftarrow C\to H_{3}$ or $H_{5}\leftarrow C\to H_{6}$) that becomes asymmetric for example, $H_{2}\to C\to H_{3}$. The interstate gradient for A/C B/C and A/B always involves the stretching of two CH bonds (NM 9 NM 11 and NM 12).

The initial vibrational motion stemming from a coherent electronic wavepacket will steer the nuclear wavepacket into a specific region of the phase space (i.e., geometry and momenta) and thus form unique “initial conditions” for the subsequent dynamics. Therefore, it is potentially possible to control the nuclear motion and the resulting photoproduct (fragmentation in C_2_H_4_^+^) by designing an initial electronic wavepacket to push the nuclear motion into a specific direction. This is the sought‐after “attocontrol” of the attochemistry field.

If the simulations are run for a longer time one might expect nuclear decoherence where the individual part of the nuclear wavepacket will travel on time‐dependent PES very close to a single adiabatic state equivalent (i.e., electronic wavepacket might decay to a single eigenstate). The speed of the nuclear decoherence will impact the extent of the photocontrol. In the case of ethylene cation, we do observe the nuclear wavepacket moving substantially along coupling motion within the 20 fs of dynamics and thus, it will have a non‐negligible impact on subsequent nuclear dynamics.

## Data Availability

The data that support the findings of this study are available from the corresponding author upon reasonable request.

## References

[1] A. Palacios and F. Martin , “The Quantum Chemistry of Attosecond Molecular Science,” WIREs Computational Molecular Science 10, no. 1 (2020): e1430, 10.1002/wcms.1430.

[2] M. Nisoli , P. Decleva , F. Calegari , A. Palacios , and F. Martin , “Attosecond Electron Dynamics in Molecules,” Chemical Reviews 117, no. 16 (2017): 10760–10825, Review, 10.1021/acs.chemrev.6b00453. 28488433

[3] F. Lepine , M. Y. Ivanov , and M. J. J. Vrakking , “Attosecond Molecular Dynamics: Fact or Fiction?,” Nature Photonics 8, no. 3 (2014): 195–204, 10.1038/Nphoton.2014.25.

[4] I. C. D. Merritt , D. Jacquemin , and M. Vacher , “Attochemistry: Is Controlling Electrons the Future of Photochemistry?,” Journal of Physical Chemistry Letters 12, no. 34 (2021): 8404–8415, 10.1021/acs.jpclett.1c02016. 34436903

[5] M. Vacher , M. J. Bearpark , and M. A. Robb , “Communication: Oscillating Charge Migration Between Lone Pairs Persists Without Significant Interaction With Nuclear Motion in the Glycine and Gly‐Gly‐NH‐CH3 Radical Cations,” Journal of Chemical Physics 140 (2014a): 201102, 10.1063/1.4879516. 24880259

[6] F. Vismarra , F. Fernández‐Villoria , D. Mocci , et al., “Few‐Femtosecond Electron Transfer Dynamics in Photoionized Donor–π–Acceptor Molecules,” Nature Chemistry 16 (2024): 2017–2024, 10.1038/s41557-024-01620-y. PMC11611723 39322782

[7] A. J. Suñer‐Rubio , C. Lemell , R. Y. Bello , J. Burgdörfer , A. Palacios , and F. Martín , “Attosecond Photoionization Delays in Molecules: The Role of Nuclear Motion,” Physical Review Research 6, no. 2 (2024): L022066, 10.1103/PhysRevResearch.6.L022066.

[8] V. Despré , A. Marciniak , V. Loriot , et al., “Attosecond Hole Migration in Benzene Molecules Surviving Nuclear Motion,” Journal of Physical Chemistry Letters 6, no. 3 (2015): 426–431, 10.1021/jz502493j. 26261959

[9] J. C. Tully , “Perspective: Nonadiabatic Dynamics Theory,” Journal of Chemical Physics 137 (2012): 22a301, 10.1063/1.4757762. 23249037

[10] J. C. Tully and R. K. Preston , “Trajectory Surface Hopping Approach to Nonadiabatic Molecular Collisions: The Reaction of H+ With D2,” Journal of Chemical Physics 55, no. 2 (1971): 562–572, 10.1063/1.1675788.

[11] M. Barbatti , G. Granucci , M. Persico , et al., “The On‐The‐Fly Surface‐Hopping Program System NEWTON‐X: Application to Ab Initio Simulation of the Nonadiabatic Photodynamics of Benchmark Systems,” Journal of Photochemistry and Photobiology A: Chemistry 190, no. 2‐3 (2007): 228–240, 10.1016/j.jphotochem.2006.12.008.

[12] M. Barbatti , “Nonadiabatic Dynamics With Trajectory Surface Hopping Method,” WIREs Computational Molecular Science 1, no. 4 (2011): 620–633.

[13] E. J. Heller , “Time‐Dependent Approach to Semiclassical Dynamics,” Journal of Chemical Physics 62, no. 4 (1975): 1544–1555, 10.1063/1.430620.

[14] T. Tran , A. Ferté , and M. Vacher , “Simulating Attochemistry: Which Dynamics Method to Use?,” Journal of Physical Chemistry Letters 15 (2024): 3646–3652, 10.1021/acs.jpclett.4c00106. 38530933 PMC11000647

[15] M. Vacher , D. Mendive‐Tapia , M. J. Bearpark , and M. A. Robb , “The Second Order Ehrenfest Method A Practical CASSCF Approach to Coupled Electron‐Nuclear Dynamics,” Theoretical Chemistry Accounts 133 (2014b): 1505, 10.1007/s00214-014-1505-6.

[16] A. Abedi , N. T. Maitra , and E. K. U. Gross , “Correlated Electron‐Nuclear Dynamics: Exact Factorization of the Molecular Wavefunction,” Journal of Chemical Physics 137 (2012): 22a530, 10.1063/1.4745836. 23249067

[17] F. Agostini , S. K. Min , A. Abedi , and E. K. U. Gross , “Quantum‐Classical Nonadiabatic Dynamics: Coupled‐ Vs Independent‐Trajectory Methods,” Journal of Chemical Theory and Computation 12, no. 5 (2016): 2127–2143, 10.1021/acs.jctc.5b01180. 27030209

[18] H. D. Meyer , U. Manthe , and L. S. Cederbaum , “The Multi‐Configurational Time‐Dependent Hartree Approach,” Chemical Physics Letters 165, no. 1 (1990): 73–78, 10.1016/0009-2614(90)87014-I.

[19] D. V. Shalashilin , “Multiconfigurational Ehrenfest Approach to Quantum Coherent Dynamics in Large Molecular Systems,” Faraday Discussions 153 (2011): 105–116, 10.1039/C1FD00034A. 22452076

[20] G. W. Richings , I. Polyak , K. E. Spinlove , G. A. Worth , I. Burghardt , and B. Lasorne , “Quantum Dynamics Simulations Using Gaussian Wavepackets: The vMCG Method,” International Reviews in Physical Chemistry 34, no. 2 (2015): 269–308, 10.1080/0144235X.2015.1051354.

[21] A. J. Jenkins and M. A. Robb , “The Damped Ehrenfest (D‐Eh) Method: Application to Non‐Adiabatic Reaction Paths,” Computational and Theoretical Chemistry 1152 (2019): 53–61, 10.1016/j.comptc.2019.02.004.

[22] J. Meisner , M. Vacher , M. J. Bearpark , and M. A. Robb , “Geometric Rotation of the Nuclear Gradient at a Conical Intersection: Extension to Complex Rotation of Diabatic States,” Journal of Chemical Theory and Computation 11, no. 7 (2015): 3115–3122, 10.1021/acs.jctc.5b00364. 26575748

[23] M. Olivucci , T. Tran , G. A. Worth , and M. A. Robb , “Unlocking the Double Bond in Protonated Schiff Bases by Coherent Superposition of S1 and S2,” Journal of Physical Chemistry Letters 12, no. 23 (2021): 5639–5643, 10.1021/acs.jpclett.1c01379. 34110826

[24] T. Tran , G. A. Worth , and M. A. Robb , “Control of Nuclear Dynamics in the Benzene Cation by Electronic Wavepacket Composition,” Communications Chemistry 4, no. 1 (2021): 48, 10.1038/s42004-021-00485-3. 36697520 PMC9814899

[25] I. Polyak , A. J. Jenkins , M. Vacher , M. E. F. Bouduban , M. J. Bearpark , and M. A. Robb , “Charge Migration Engineered by Localisation: Electron‐Nuclear Dynamics in Polyenes and Glycine,” Molecular Physics 116 (2018): 2474–2489, 10.1080/00268976.2018.1478136.

[26] D. Danilov , T. Tran , M. J. Bearpark , J. P. Marangos , G. A. Worth , and M. A. Robb , “How Electronic Superpositions Drive Nuclear Motion Following the Creation of a Localized Hole in the Glycine Radical Cation,” Journal of Chemical Physics 156 (2022): 244114, 10.1063/5.0093780. 35778090

[27] M. Deumal , J. Ribas‐Ariño , and M. A. Robb , “Using “Designer” Coherences to Control Electron Transfer in a Model Bis(Hydrazine) Radical Cation: Can We Still Distinguish Between Direct and Superexchange Mechanisms?,” Journal of Physics B: Atomic, Molecular and Optical Physics 57 (2024): 075001.

[28] G. A. Worth and M. A. Robb , “Controlling Electronic Coherences and the Curvature Induced by the Derivative Coupling at a Conical Intersection: A Quantum Ehrenfest (QuEh) Protocol for Reaction Path Following Application to “Channel 3” Benzene Photochemistry,” Journal of Physical Chemistry A 128, no. 27 (2024): 5408–5415, 10.1021/acs.jpca.4c02449. 38917388 PMC11247493

[29] C. S. M. Allan , B. Lasorne , G. A. Worth , and M. A. Robb , “A Straightforward Method of Analysis for Direct Quantum Dynamics: Application to the Photochemistry of a Model Cyanine,” Journal of Physical Chemistry A 114, no. 33 (2010): 8713–8729. 20499843 10.1021/jp101574b

[30] B. G. Levine , J. D. Coe , A. M. Virshup , and T. J. Martínez , “Implementation of Ab Initio Multiple Spawning in the Molpro Quantum Chemistry Package,” Chemical Physics 347, no. 1 (2008): 3–16, 10.1016/j.chemphys.2008.01.014.

[31] A. Gaenko , A. DeFusco , S. A. Varganov , T. J. Martinez , and M. S. Gordon , “Interfacing the Ab Initio Multiple Spawning Method With Electronic Structure Methods in GAMESS: Photodecay of Trans‐Azonnethane,” Journal of Physical Chemistry A 118, no. 46 (2014): 10902–10908, 10.1021/jp508242j. 25329724

[32] S. Mattos , S. Mukherjee , and M. Barbatti , “Quantum Dynamics From Classical Trajectories,” Journal of Chemical Theory and Computation 20, no. 18 (2024): 7728–7743, 10.1021/acs.jctc.4c00783. 39235064

[33] P. A. M. Dirac , “Note on Exchange Phenomena in the Thomas Atom,” Proceedings of the Cambridge Philosophical Society 26 (1930): 376–385, 10.1017/S0305004100016108.

[34] R. Stockbauer and M. G. Inghram , “Threshold Photoelectron–Photoion Coincidence Mass Spectrometric Study of Ethylene and Ethylene‐d4,” Journal of Chemical Physics 62, no. 12 (1975): 4862–4870, 10.1063/1.430398.

[35] J. E. Pollard , D. J. Trevor , J. E. Reutt , Y. T. Lee , and D. A. Shirley , “Torsional Potential and Intramolecular Dynamics in the C2H+4 Photoelectron Spectra,” Journal of Chemical Physics 81, no. 12 (1984): 5302–5309, 10.1063/1.447672.

[36] S. Willitsch , U. Hollenstein , and F. Merkt , “Ionization From a Double Bond: Rovibronic Photoionization Dynamics of Ethylene, Large Amplitude Torsional Motion and Vibronic Coupling in the Ground State of C2H4+,” Journal of Chemical Physics 120, no. 4 (2004): 1761–1774, 10.1063/1.1635815. 15268306

[37] J. C. Lorquet , C. Sannen , and G. Raseev , “Dissociation of the Ethylene Cation: Mechanism of Energy Randomization,” Journal of the American Chemical Society 102, no. 27 (1980): 7976–7977, 10.1021/ja00547a045.

[38] C. Sannen , G. Raşeev , C. Galloy , G. Fauville , and J. C. Lorquet , “Unimolecular Decay Paths of Electronically Excited Species. II. The C2H+4 Ion,” Journal of Chemical Physics 74, no. 4 (1981): 2402–2411, 10.1063/1.441361.

[39] M. H. Kim , B. D. Leskiw , and A. G. Suits , “Vibrationally Mediated Photodissociation of Ethylene Cation by Reflectron Multimass Velocity Map Imaging,” Journal of Physical Chemistry A 109, no. 35 (2005): 7839–7842, 10.1021/jp053143m. 16834162

[40] M. H. Kim , B. D. Leskiw , L. Shen , and A. G. Suits , “Vibrationally Mediated Photodissociation of C2H4+,” Journal of Physical Chemistry A 111, no. 31 (2007): 7472–7480, 10.1021/jp071348k. 17622124

[41] B. Joalland , T. Mori , T. J. Martínez , and A. G. Suits , “Photochemical Dynamics of Ethylene Cation C2H4+,” Journal of Physical Chemistry Letters 5, no. 8 (2014): 1467–1471, 10.1021/jz500352x. 26269995

[42] J. V. Tilborg , T. K. Allison , T. W. Wright , et al., “Femtosecond Isomerization Dynamics in the Ethylene Cation Measured in an EUV‐Pump NIR‐Probe Configuration,” Journal of Physics B: Atomic, Molecular and Optical Physics 42, no. 8 (2009): 081002, 10.1088/0953-4075/42/8/081002.

[43] A. Ludwig , E. Liberatore , J. Herrmann , et al., “Ultrafast Relaxation Dynamics of the Ethylene Cation C2H4+,” Journal of Physical Chemistry Letters 7, no. 10 (2016): 1901–1906, 10.1021/acs.jpclett.6b00646. 27139223

[44] K. S. Zinchenko , F. Ardana‐Lamas , I. Seidu , et al., “Sub‐7‐Femtosecond Conical‐Intersection Dynamics Probed at the Carbon K‐Edge,” Science 371, no. 6528 (2021): 489–494, 10.1126/science.abf1656. 33510022

[45] M. Vacher , A. Boyer , V. Loriot , F. Lépine , and S. Nandi , “Few‐Femtosecond Isotope Effect in Polyatomic Molecules Ionized by Extreme Ultraviolet Attosecond Pulse Trains,” Journal of Physical Chemistry A 126, no. 34 (2022): 5692–5701, 10.1021/acs.jpca.2c03487. 35994358

[46] M. Lucchini , B. Mignolet , M. Murari , et al., “Few‐Femtosecond C2H4+ Internal Relaxation Dynamics Accessed by Selective Excitation,” Journal of Physical Chemistry Letters 13, no. 48 (2022): 11169–11175, 10.1021/acs.jpclett.2c02763. 36445180 PMC9937561

[47] L. Fransén , T. Tran , S. Nandi , and M. Vacher , “Dissociation and Isomerization Following Ionization of Ethylene: Insights From Nonadiabatic Dynamics Simulations,” Journal of Physical Chemistry A 128, no. 8 (2024): 1457–1465, 10.1021/acs.jpca.3c06512. 38358308 PMC10911106

[48] F. Agostini and B. F. E. Curchod , “When the Exact Factorization Meets Conical Intersections,” European Physical Journal B 91, no. 7 (2018): 141, 10.1140/epjb/e2018-90117-6.

[49] M. Cardosa‐Gutierrez , R. D. Levine , and F. Remacle , “Electronic Coherences Built by an Attopulse Control the Forces on the Nuclei,” Journal of Physics B: Atomic, Molecular and Optical Physics 57, no. 13 (2024): 133501, 10.1088/1361-6455/ad4fd3.

[50] J. Janoš , P. Slavíček , and B. F. E. Curchod , “Including Photoexcitation Explicitly in Trajectory‐Based Nonadiabatic Dynamics at no Cost,” Journal of Physical Chemistry Letters 15, no. 42 (2024): 10614–10622, 10.1021/acs.jpclett.4c02549. 39405399 PMC11514012

[51] M. Ruberti , P. Decleva , and V. Averbukh , “Full Ab Initio Many‐Electron Simulation of Attosecond Molecular Pump–Probe Spectroscopy,” Journal of Chemical Theory and Computation 14, no. 10 (2018): 4991–5000, 10.1021/acs.jctc.8b00479. 30180561

[52] A. Jenkins , K. Spinlove , M. Vacher , G. Worth , and M. Robb , “The Ehrenfest Method With Fully Quantum Nuclear Motion (Qu‐Eh): Application to Charge Migration in Radical Cations,” Journal of Chemical Physics 149, no. 9 (2018): 094108. 30195291 10.1063/1.5038428

[53] K. E. Spinlove , M. Vacher , M. Bearpark , M. A. Robb , and G. A. Worth , “Using Quantum Dynamics Simulations to Follow the Competition Between Charge Migration and Charge Transfer in Polyatomic Molecules,” Chemical Physics 482 (2017): 52–63, 10.1016/j.chemphys.2016.10.007.

[54] G. A. Worth , G. W. Richings , I. Burghardt , M. H. Beck , A. Jäckle , and H.‐D. Meyer , The QUANTICS Package (Birmingham, U.K: University of Birmingham, 2020).

[55] M. J. Frisch , H. B. Schlegel , G. E. Scuseria , et al., Gaussian Development Version J.05, Gaussian, Inc (Wallingford, CT: Gaussian, Inc., 2019).

[56] L. S. Cederbaum and J. Zobeley , “Ultrafast Charge Migration by Electron Correlation,” Chemical Physics Letters 307, no. 3 (1999): 205–210, 10.1016/S0009-2614(99)00508-4.

